# Identification of potentially pathogenic variants for autism spectrum disorders using gene-burden analysis

**DOI:** 10.1371/journal.pone.0273957

**Published:** 2023-05-11

**Authors:** Nika Rihar, Danijela Krgovic, Nadja Kokalj-Vokač, Spela Stangler-Herodez, Minja Zorc, Peter Dovc

**Affiliations:** 1 Biotechnical Faculty, Department of Animal Science, University of Ljubljana, Ljubljana, Slovenia; 2 Laboratory of Medical Genetics, University Medical Centre Maribor, Maribor, Slovenia; 3 Maribor Medical Faculty, University of Maribor, Maribor, Slovenia; National Institute of Child Health and Human Development (NICHD), NIH, UNITED STATES

## Abstract

Gene- burden analyses have lately become a very successful way for the identification of genes carrying risk variants underlying the analysed disease. This approach is also suitable for complex disorders like autism spectrum disorder (ASD). The gene-burden analysis using Testing Rare Variants with Public Data (TRAPD) software was conducted on whole exome sequencing data of Slovenian patients with ASD to determine potentially novel disease risk variants in known ASD-associated genes as well as in others. To choose the right control group for testing, principal component analysis based on the 1000 Genomes and ASD cohort samples was conducted. The subsequent protein structure and ligand binding analysis usingI-TASSER package were performed to detect changes in protein structure and ligand binding to determine a potential pathogenic consequence of observed mutation. The obtained results demonstrate an association of two variants–p.*Glu*198*Lys* (PPP2R5D:c.592G>A) and p.*Arg*253*Gln* (PPP2R5D:c.758G>A) with the ASD. Substitution p.*Glu*198*Lys* (PPP2R5D:c.592G>A) is a variant, previously described as pathogenic in association with ASD combined with intellectual disability, whereas p.*Arg*253*Gln* (PPP2R5D:c.758G>A) has not been described as an ASD-associated pathogenic variant yet. The results indicate that the filtering process was suitable and could be used in the future for detection of novel pathogenic variants when analysing groups of ASD patients.

## Introduction

Autism spectrum disorders (ASDs) are classified as pervasive developmental disorders. The diagnosis of ASDs is based on criteria in two areas: a deficit in social communication and interaction, and the presence of limited, repetitive patterns of behaviour, interests, and activities. Symptoms should be present early in development but may become apparent also later when social demands exceed the patient’s limited abilities [1]. ASDs are divided into non-syndromic and syndromic forms. In the former, ASD is considered the main diagnosis, while in the latter it is only one part of a complex disorder that may also include other developmental abnormalities [2]. In this complex disorder, both, genetics and environment play an important role, as well as the interaction between them [3]. The prevalence of ASD is estimated to be 1–2 per 100 [4]. Since recent studies estimate the heritability of the disorder to be 0.84–0.90, many researchers have focused on performing genomic analyses of individuals with ASD [5–7]. Their main goal is to discover genes and biological pathways associated with the disorder that should explain the pathogenesis, allow for better diagnosis, and enable the use of potential new agents in treatment [8,9]. However, gene and pathway discovery is a major challenge because patients diagnosed with ASD have very heterogeneous phenotypes and causal loci that contribute to their phenotype [10]. Genes that have been associated with ASD, play important roles in chromatin remodelling [11–14], protein synthesis and ubiquitination [13], and synapse [13,14] and neuron [14] development and function. Variants in these ASD-associated genes are both, common and rare [11,13,15,16]. It is suggested that much of the risk for the disorder can be attributed to the rare variants with strong effects [17]. Rare copy number variations (CNVs) play an important role in susceptibility to the disorder. The most important CNV associated with ASD is the 16p11.2 deletion, which has also been associated with other psychiatric disorders [18,19]. In addition to CNVs, rare point variants also increase the risk for developing the disorder [20,21].

The study of these rare variants in exome sequencing data using gene-burden analyses has recently become a very attractive and effective way to identify disease associated genes [22]. These gene-burden analyses have also been useful for the discovery of genes in complex disorders such as ASD, which have high heterogeneity of causal genes [23]. The basic concept of gene-burden analysis is to compare the number of likely pathogenic variants in the selected cases versus controls in each of the genes studied [24].

The most challenging task in the process of analysis is the detection and selection of likely disease-causing variants, which are called qualifying variants. The so-called qualifying variants are then further examined using appropriate filters, such as various quality metrics, allele frequency, and predicted consequence and pathogenicity using different tools [25]. Gene-burden analysis can be performed using local, in-house software, but it is also possible to use available software from other sources. Software packages that allow gene-burden testing using public databases (ExAC, GnomAD [26]) have proven to be a useful tool for association of disease-associated genes in cases, where no control cohort is available. For example, Testing Rare Variants using Public Data (TRAPD) allows custom selection of all possible minor allele frequencies (MAF), subcategories of populations (e.g. non-Finnish Europeans), filtering of variants based on annotations (PolyPhen [27], SIFT [28], Mutation Taster [29]) and selection of any control population [30]. TRAPD has been used to successfully discover disease-associated genes associated with various diseases such as Diamond-Blackfan anemia, human spiradenoma, spiradenocarcinoma, Meniere’s disease, and amyotrophic lateral sclerosis [31–35].

Burden testing for ASD has been done a few times [36–38]. In smaller studies, the number of individuals tested has been a limitation to the discovery of ASD-associated genes [36,37]. To increase the possibility of discovering new associations, some studies used larger test cohorts [38]. Stricter criteria for variant and gene selection (only truncating variants in a haploinsufficient gene that has no truncating variation in the healthy control population) also proved to be a successful way to discover associations [38]. The results show that due to the highly polygenic nature of the disease, large test cohorts and pre-selection of genes suitable for testing are necessary [36–38].

Considering all the advantages and disadvantages of the above-mentioned studies, we believe that by using large control cohorts from large databases, such as the Genome Aggregation Database (GnomAD), reducing phenotypic heterogeneity by including only patients with similar phenotypes, and selecting likely pathogenic variants and tested genes according to more stringent criteria, the association of genes with pathogenic variants underlying ASD could be discovered. The main objective of our study was to analyse whole-exome sequencing data from 35 Slovenian patients diagnosed with ASD in combination with intellectual disability (ID) followed by the application of gene-burden testing using TRAPD software and a control cohort from the GnomAD database. In addition, our study aims to discover novel ASD-associated probably pathogenic variants and thus contribute to clinical knowledge on variant pathogenicity using a combination of allele frequencies in the population, pathogenicity prediction tools, o/e-, and Z scores for variant and gene selection for burden testing. The aim of this study was also to identify genes underlying the disease in the Slovenian population.

## Materials and methods

### Autism spectrum disorders cohort

The Laboratory of medical genetics at the University Medical Centre Maribor, Slovenia, collected DNA from 99 Slovenian subjects diagnosed with ASD who had been referred to the laboratory as part of a standard diagnostic procedure. This experiment was approved by the University Medical Centre of Maribor and the Ethics committee. Written informed consent to participate in this study was provided by all participants or their legal guardians. Their DNA was obtained from whole blood samples.

### Whole exome sequencing

Whole exome sequencing analysis of 99 samples was performed by Novogene (China). The starting material for sample preparation was 1.0 μg genomic DNA per sample. Sequencing libraries were generated using Agilent SureSelect Human All ExonV6 kit (Agilent Technologies, CA, USA) according to the manufacturer recommendations. Index codes were added to distinguish samples after pooling. The main steps of library preparation were performed as follows. First, DNA was sheared into 180–280 bp fragments using hydrodynamic shearing system (Covaris, Massachusetts, USA). The remaining overhangs were converted to blunt ends by exonuclease/polymerase activity. This step was followed by the adenylation of the 3’ ends of the DNA fragments and ligation of adapter oligonucleotides. Only the fragments that were ligated with adapters at both ends were then enriched by polymerase chain reaction. Index tags were then added, followed by hybridization of the samples. After hybridization, the products were purified using the AMPure XP system (Beckman Coulter, Beverly, USA). Quantification before pooling, according to the sample concentration was performed using the Agilent High Sensitivity DNA Assay on the Agilent Bioanalyzer 2100 system. Finally, the pooled libraries were loaded onto Illumina HiSeq 2500 sequencer.

### Variant calling and annotation

**Fig 1:**
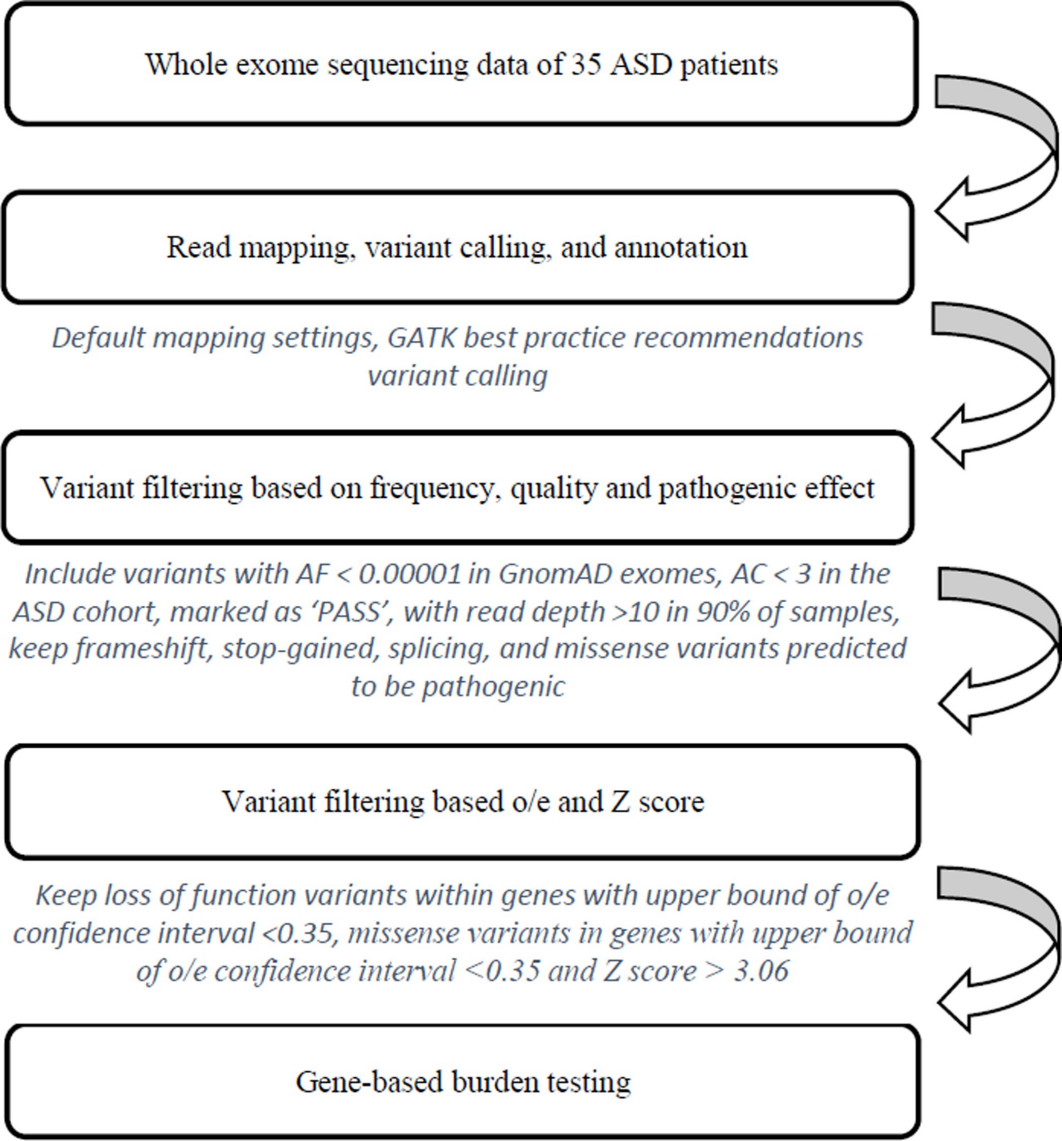
Flow chart for variant detection and prioritizing for gene-burden testing.

The generated sequencing reads (fastq file format) were aligned to the human reference genome build b37 using BWA software version 0.7.10 [39]. Subsequent bioinformatic analyses of the binary bam files were performed using Genome Analysis Toolkit toolset version 3.5 according to best practice recommendations for variant calling, such as marking duplicates, quality score recalibration of bam files, and variant calling with HaplotypeCaller [40]. Subsequently, the variant call format (VCF) files of all patients were combined into one VCF file and then analysed using VariantRecalibrator for variant quality recalibration, which assigns a probability score for a true genetic variant to each variant in a VCF file. Finally, the variant call file was annotated using Variant Effect Predictor v98 (Fig 1) [41].

### Principal component analysis

Principal Component Analysis (PCA) of common variants was performed on exome regions of 1000 Genomes Phase 3 and ASD cohort data. SNPs were pruned using PLINK 1.9, based on linkage disequilibrium [42]. The variance inflation threshold of 1.5 was applied, and variants with minor allele frequencies greater than 10% were retained in the analysis. R v3.5.2 software was used for graphical representation [43]. The ancestry of the ASD cohort was predicted using principal component coordinates.

### Reduction of phenotypic heterogeneity

To reduce phenotype heterogeneity, only unrelated patients with the ASD phenotype in combination with ID from the ASD cohort were included in the following gene load analysis. This group of 35 patients was selected based on the fact that ID is most influenced by genetics and the least by environment of all neurodevelopmental disorders [44]. In addition, the underlying genetic cause of the disorder is more frequently discovered in patients with a combined ID diagnosis [45].

### Gene-burden analysis

Gene-burden testing between case (35 unrelated case subjects with a diagnosis of ASD combined with ID from the ASD cohort) and control subjects was performed using TRAPD software. Because the case subjects belong to the group of Europeans of non-Finnish descent and have neurological phenotypes, the non-Finnish non-neurological group of control subjects from GnomAD exomes v2.1 was used for testing. GnomAD exomes v2.1 is an exome sequencing aggregation database of 125,748 individuals, not known to have a severe Mendelian disease. Variants with a MAF of less than 0.001% in the GnomAD database or that were not present in the GnomAD database were considered rare and included in the analysis. In order to also consider the variants that are common in the Slovenian population and not present in the GnomAD exome database, the variants with an allele count of less than 3 in the ASD cohort were considered rare in Slovenian population and were also considered in the further analysis. Considering the already published results we decided to focus only on very rare variants [25,46–50]. In the process of burden testing, rare synonymous variants were first analysed to adjust the sequencing quality thresholds and to ensure that there was no significant enrichment of genes. To ensure uniform sequencing coverage of the analysed variants and thus avoid biased results, only variants at sites where the sequencing depth was greater than 10x in more than 90% of the samples were retained. Next, among the possible quality scores quality by depth (QD), Phred quality (QUAL), genotype Phred quality (GQ), mapping quality (MQ), and variant quality score log-odds (VQSLOD) scores [22,24,30], were selected for variant filtering. Only variants marked as “PASS” based on the VQSLOD were further analysed. After establishing appropriate quality thresholds, a burden analysis was performed for rare stop-gains, frameshifts, splice acceptors, splice donors, and missense which were classified as ‘probably damaging’ by PolyPhen-2, ‘deleterious’ by SIFT, the likelihood ratio test (LRT) and RadialSVM, ‘disease-causing’ by MutationTaster and ‘high’ by MutationAssessor [29,51–53]. In gene-burden analyses, variants that have a greater impact on proteins (frameshift, stop-gain, splicing), and occasionally those that have a lesser impact on them (missense variants) are usually analysed [30,35,48]. The decision to focus on both missense and loss-of-function variants (frameshift, stop-gain, splicing) was based on the assumption that both categories can be pathogenic. To analyse only the more deleterious missense variants, the results of pathogenicity prediction tools (Polyphen-2, LRT, MutationTaster, MutationAssessor, RadialSVM and SIFT) were considered. Prior to analysis, all selected variants were also filtered based on the observed/expected (o/e) score and the Z score of a gene. This allowed us to adequately test scores for gene intolerance to variation based on analyses of large healthy control populations such as the GnomAD. The o/e ratio is the ratio between the number of observed variants and the number of expected variants with loss of function. It is strongly recommended that haploinsufficient genes should be selected using the upper limit of the confidence interval of less than 0.35 [26]. Another value, developed by researchers is the Z score, which indicates the tolerance of a gene to missense variation, with higher Z scores indicating lower tolerance to this category of variation [54]. Considering the above recommendations, loss-of-function variants were retained in genes with an upper limit of the o/e confidence interval of less than 0.35 and missense variants were retained in haploinsufficient genes also in the top 5% when the Z score was considered (Fig 1). The o/e and Z scores, used were taken from the GnomAD table ‘pLoF Metrics by Gene’ (https://gnomad.broadinstitute.org/downloads).

#### Analysis of the target region

To validate the target region by Sanger sequencing, two primer pairs were designed for polymerase chain reaction (PCR). To validate the p.*Arg*253*Gln* mutation, primers 5’-AGAGGAAGATGAGCCCACCC-3’ and 5’-CTGCCTACGGATATAAGCCCG-3’ were used to amplify a 785 bp long product. The region containing the p.*Glu*198*Lys* mutation was amplified with primers 5’-CACCCATAGCCGTGATGTTGT-3’ and 5’-GAGCAAGTACAAACTTCTGGTCG-3’, resulting in a 452 bp long product. A 25 μl PCR reaction contained 17 μl nuclease-free water, 5 μl 5X One Taq Standard Reaction Buffer (NEB), 5 pmol of each primer, 0.2 mmol/l deoxynucleoside triphosphates (dNTPs) (Thermo Scientific), 0.6 U One*Taq* DNA Polymerase (NEB), and 50 ng DNA. Reaction conditions were as follows: 94°C 30s; 30 cycles 94°C 30s, 61°C 30s, 68°C 1 min; 68°C 5 min. PCR products were then sequenced on the Applied Biosystems 3500 genetic analyser, using primers 5’-CATCACTTTGGAAGTCTCAGTACAA-3’ and 5’-CACCCATAGCCGTGATGTTGT-3’ for validation of the p.*Arg*253*Gln* and p.*Glu*198*Lys* variants, respectively.

The p.*Arg*253*Gln* mutation was also validated by restriction fragment length polymorphism (RFLP) analysis. For PCR, primers 5’-TGAGGCATCACTTTGGAAGTCT-3’ and 5’-GCTCTCTTGACAACCCCTGA-3’ were used to amplify a 442 bp long product. The reaction mixture was the same as described previously and the conditions were as follows: 94°C 30s; 30 cycles 94°C 30s, 59°C 30s, 68°C 1 min; 68°C 5 min. Then, nested PCR was performed with the PCR product using primers 5’-CAGGGGACCTCTGCATTTC-3’ and 5’-GCTCTCTTGACAACCCCTGA-3’ under the following conditions: 94°C 30s; 30 cycles 94°C 30s, 58°C 30s, 68°C 1 min; 68°C 5 min. A 25 μl reaction contained 1 μl of a 10^−1^ dilution of PCR product, 17 μl nuclease-free water, 5 μl 5X One Taq Standard Reaction Buffer, 5 pmol of each primer, 0,2 mmol/l dNTPs, and 0,6 U One*Taq* DNA Polymerase. This yielded a 351 bp long product, that was digested with *Xho*I. The 7 μl reaction contained 4 μl H_2_O, 2 μl of the amplification product, 0.7 μl 10X NEB buffer and 10 U enzyme and was incubated at 37°C for 3 h. The digestion products were separated on a 2% agarose gel and stained with ethidium bromide.

### Prediction of protein structure and function

Software I-TASSER was used to predict the effects of the p.*Arg*253*Gln* mutation on protein structure and function. The original and modified amino-acid sequences of PPP2R5D were analysed on the online server I-TASSER [55,56].

## Results

### Genetic ancestry of Slovenian ASD patients

**Fig 2:**
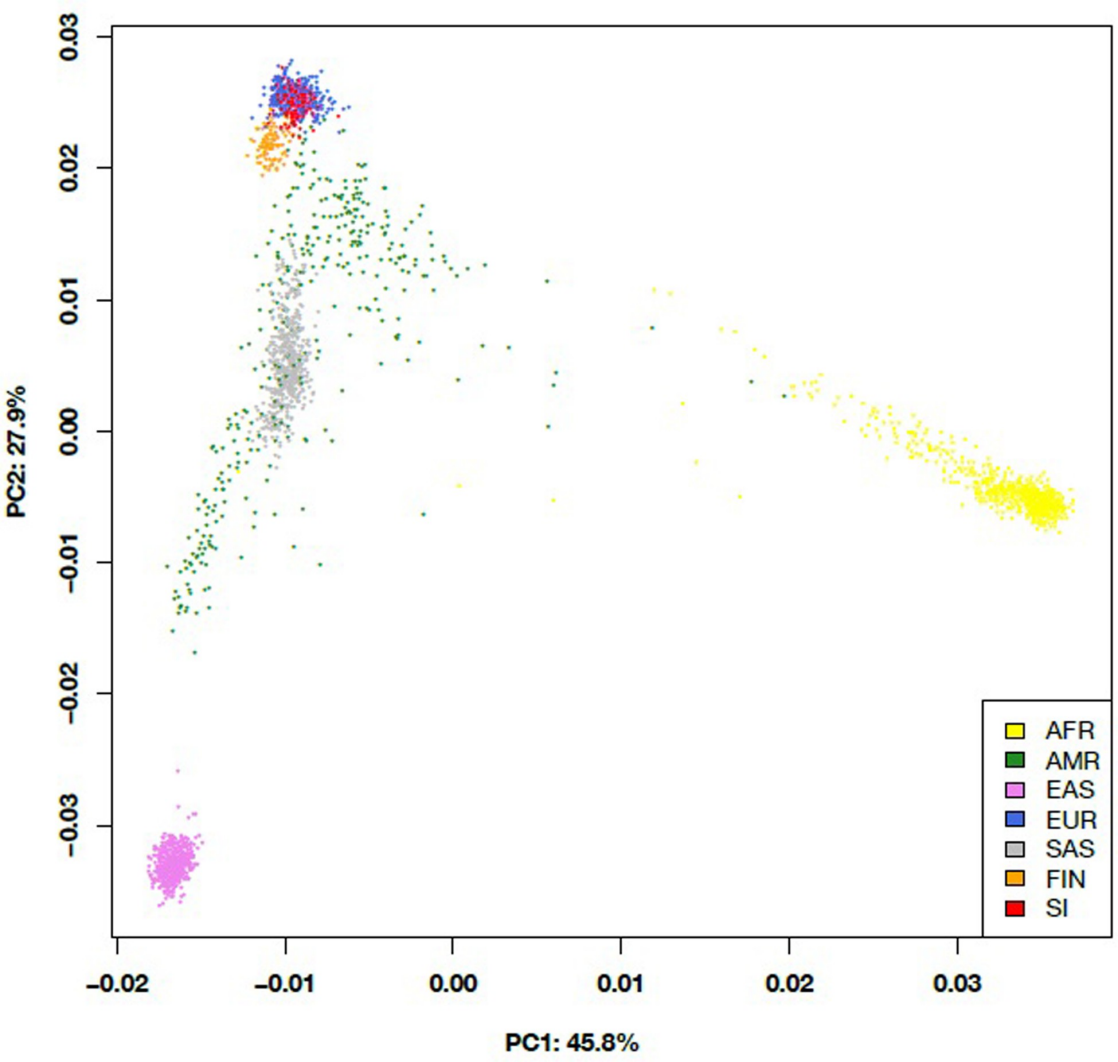
PCA based on the 1000 Genomes and ASD cohort samples.

The individuals from the 1000 Genomes Project and the Slovenian ASD patients were plotted based on the first two principal components. The data of all individuals are coloured according to their superpopulation (AFR—African, AMR—Admixed American, EAS–East Asian, EUR—European, SAS–South Asian), except for the Finnish (FIN) and Slovenian (SI) ASD patients who are coloured in their own colours. The Slovenian patients are positioned at the position of the Europeans, while the Finnish population is positioned next to the sample data of the Europeans. Considering these principal component coordinates, the cohort of ASD patients belongs to the non-Finnish European population (Fig 2).

### Significant genes in autism spectrum disorders

**Fig 3:**
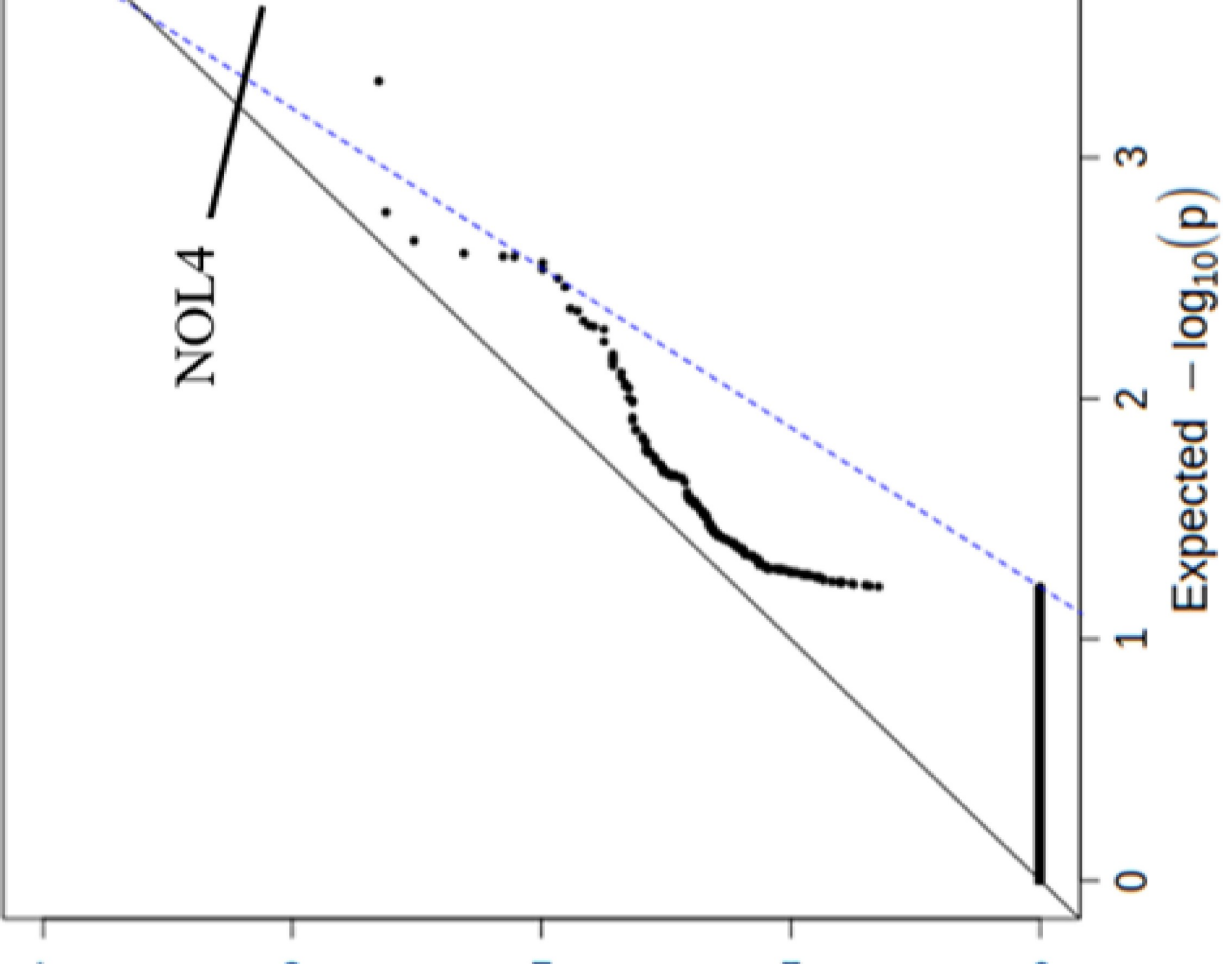
Quantile-quantile plot showing the–log_10_ of p values of the burden testing considering only synonymous, rare variants versus the expected–log_10_ of p values. Values are given for genes (black dots). The black line represents the ratio between expected and observed p values when the distribution of p values is uniform, and the blue, dotted line represents the actual ratio when genes are considered that fall in the range between the 50^th^ and 90^th^ percentile. The most significantly associated gene is *NOL4*.

**Fig 4:**
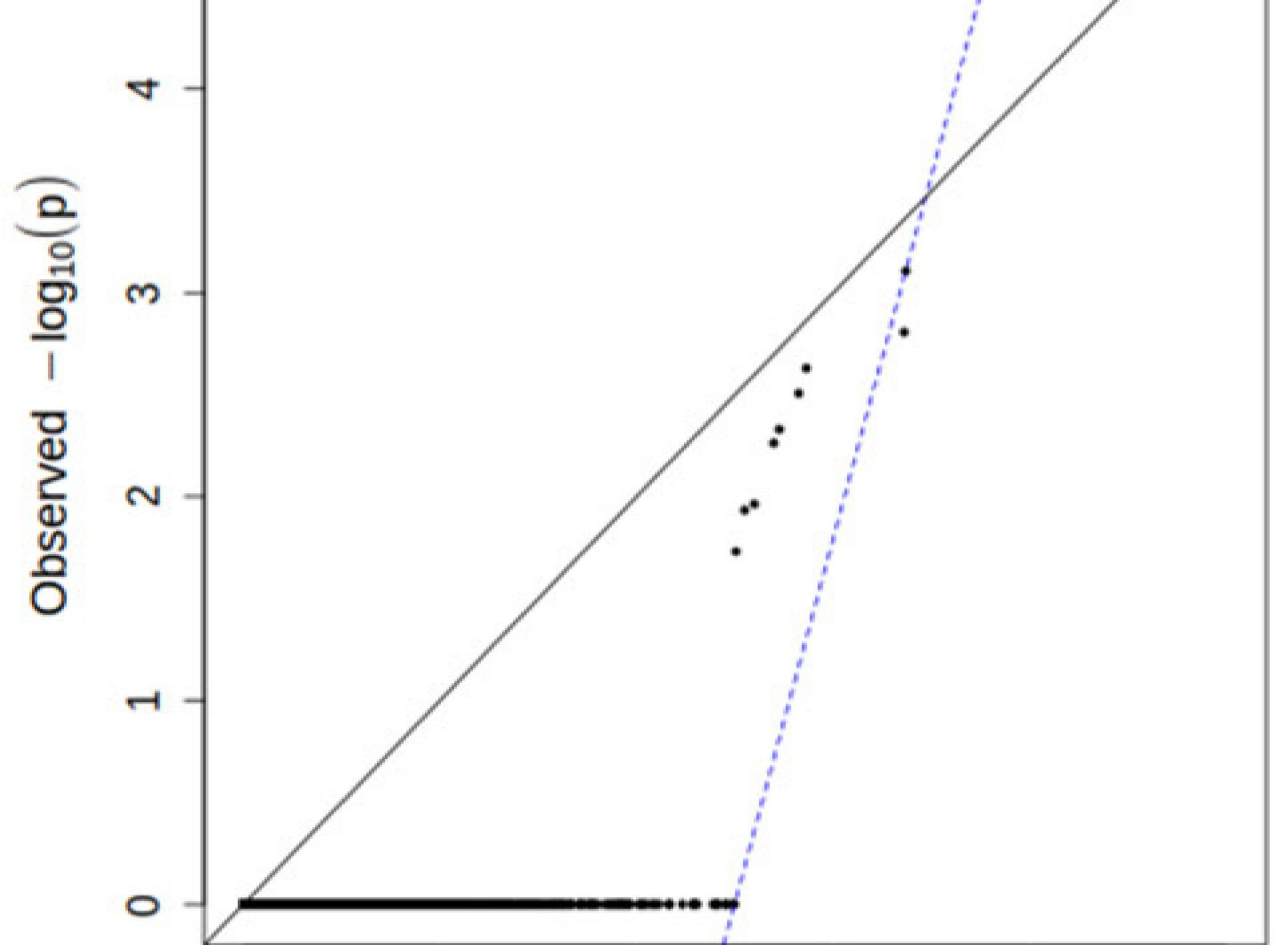
The results of burden testing for rare, probable pathogenic mutations. PPP2R5D is the most significantly associated gene, and has more mutations than expected.

The results of the rare synonymous burden analysis showed no significant enrichment of genes, enriched in the following analysis of rare, probably pathogenic variants. The most strongly associated gene was NOL4, which, however, contained fewer variants than expected. Some of the genes have no variants in the population of ASD patients, representing a narrow line along the x-axis (Fig 3). Since the number of genes in the genome is about 19000, the significance exome wide threshold α is after correction for multiple testing (0.05/19000) set to 2.6 × 10^−6^ [30,35]. Because the number of genes included in this burden analysis was 2735, the experiment wide threshold α was set at 1.8 × 10^−5^ (0.05/2735). After applying the burden testing of pathogenic variants on these genes, an experiment wide threshold association of *PPP2R5D* gene (p = 1.7 × 10^−5^) was identified. Other genes were less significantly associated. Many genes in the group of ASD patients did not have potentially pathogenic variants, as shown by the dots forming a horizontal line along to the x-axis (Fig 4). Two patients in our cohort were found to have a risk missense mutation in this gene. One of these patients had a mutation p.*Arg253Gln* and the other one a mutation p.*Glu*198*Lys*. One of the patients was diagnosed with ID and suspected ASD, while the other was diagnosed with autism and ID (Table 1).

**Table 1:** Phenotypes of individuals with pathogenic variants in PPP2R5D.

Individual	1	2
Variant	p.*Arg*253*Gln*	p.*Glu*198*Lys*
Phenotype	ID and suspected ASD	autism and ID

### Analysis of the target region

**Fig 5:**
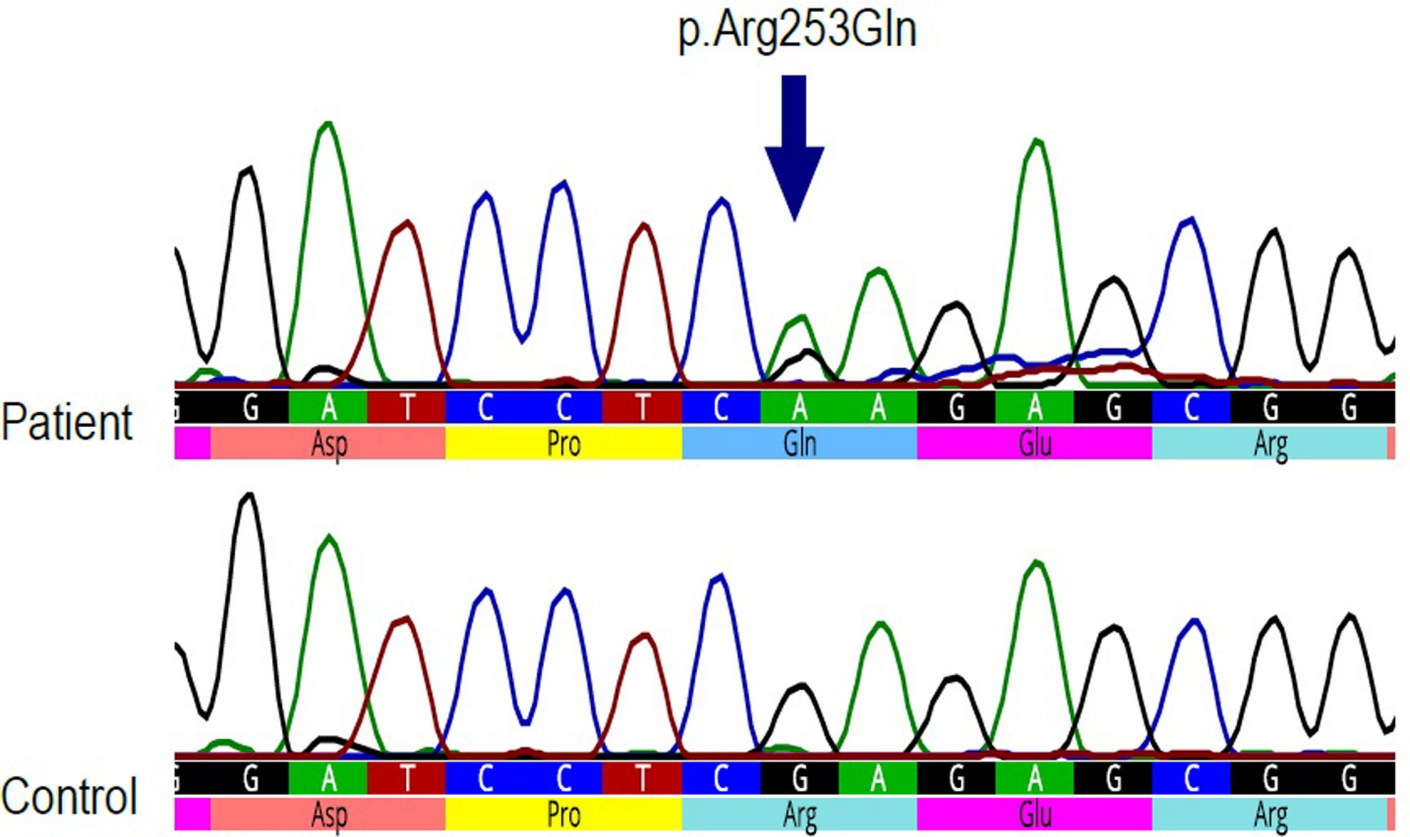
Sanger sequencing results for variant p.*Arg*253*Gln*. On the left side is the sequence of a person without mutation and on the right side is the sequence of the patient heterozygous for p.*Arg*253*Gln* mutation.

**Fig 6:**
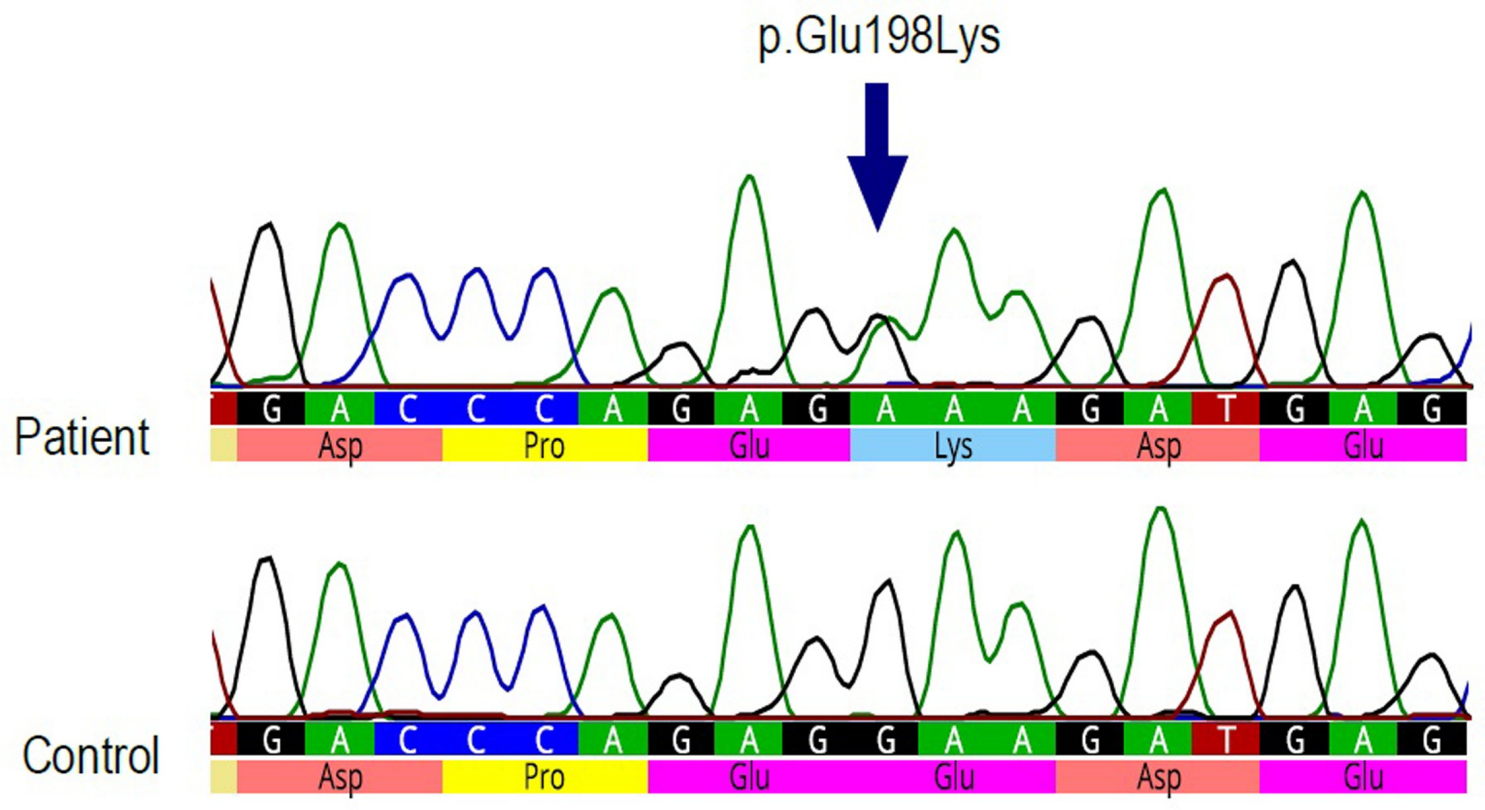
Sanger sequencing results for variant p.*Glu*198*Lys*. On the left side is the sequence of a person without mutation and on the right side is the sequence of the patient heterozygous for p.*Glu*198*Lys* mutation.

**Fig 7:**
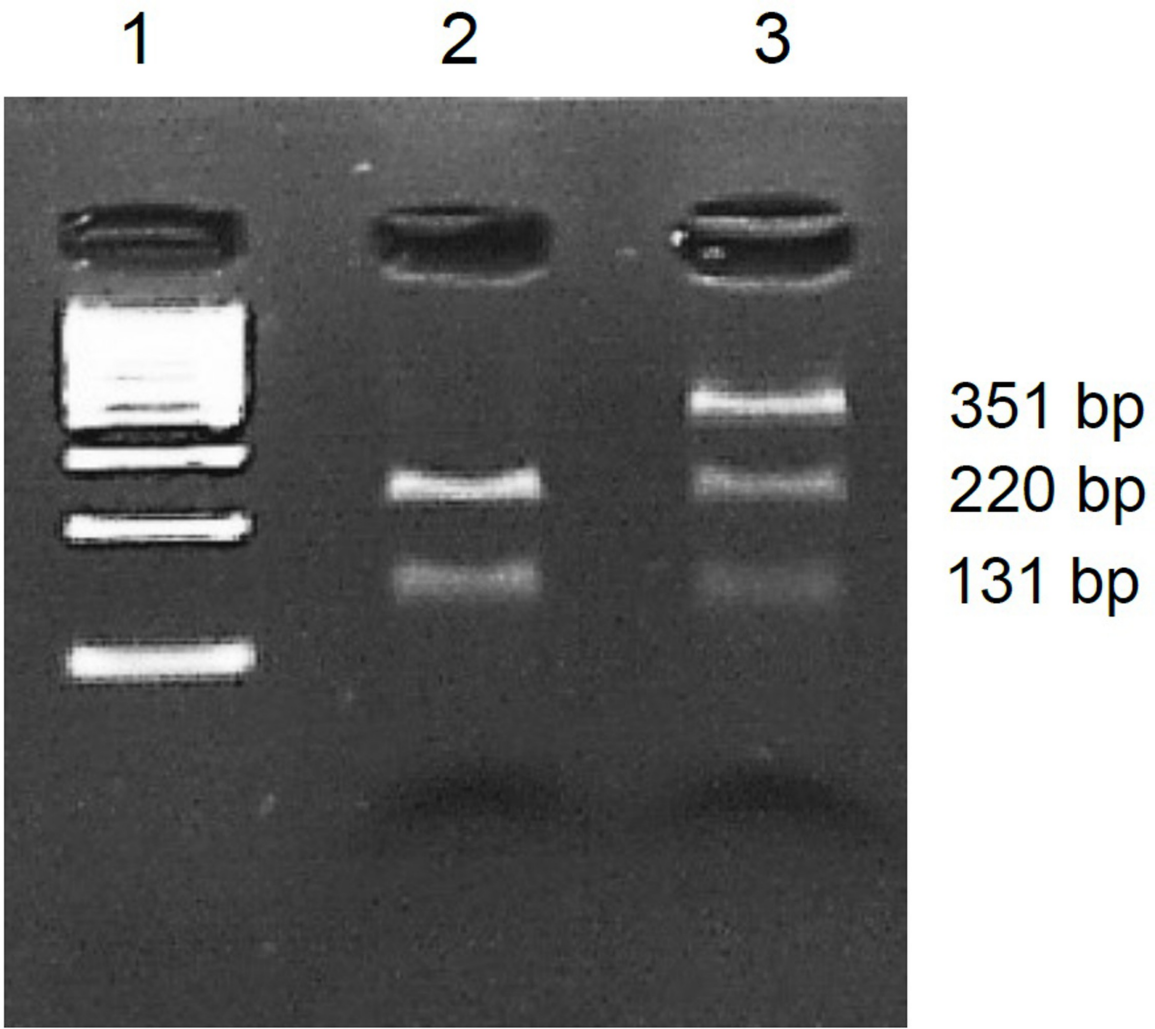
Results of PCR-RFLP analysis of p.*Arg*253*Gln* mutation. Lane 1: 100 bp DNA ladder, lane 2: A person without mutation, lane 3: The patient, heterozygous for p.*Arg*253*Gln* mutation.

Both variants, p.*Arg*253*Gln* (Fig 5) and p.*Glu*198*Lys* (Fig 6) were confirmed by Sanger sequencing. The p.*Arg*253*Gln* variant was also confirmed by RFLP. The restriction enzyme *Xho*I cut the PCR product without mutation into two fragments, whereas it did not cut the product with mutation. The heterozygous mutation was identified by the presence of an undigested, 351 bp long fragment, and the digested 220 bp and 131 bp long fragments (Fig 7).

### Prediction of ligand binding sites on PPP2R5D

**Fig 8:**
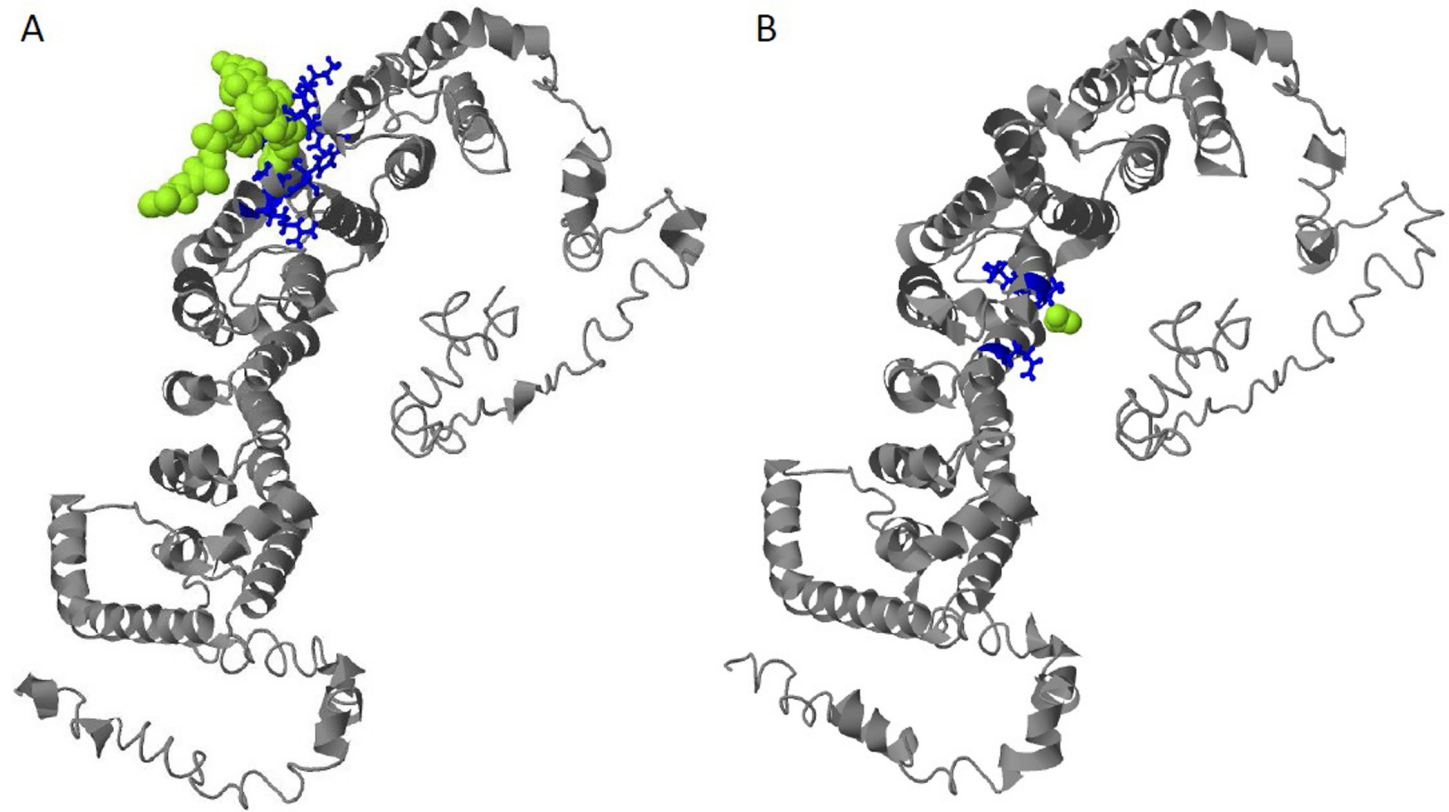
Results of ligand binding site prediction for the original (A) and mutant (B) protein sequences. Software I-TASSER suggests the ligand most likely to bind to the analysed protein. The binding protein residues are shown in blue, and the predicted binding ligands are shown in green-yellow.

The results of predicting the consequences of the p.*Arg*253*Gln* mutation on protein structure and function show that the possible ligand, Importin subunit beta-1, that binds to the original protein (Fig 8A), is not a plausible ligand for the mutant protein (Fig 8B). These I-TASSER results of protein-ligand binding sites are based on structure comparison, protein-protein networks, and detection of ligand binding templates.

## Discussion

Analysis of rare, probably pathogenic variants, revealed an association with two risk variants. Both variants are located in the *PPP2R5D* gene, which plays an important role in the regulation of neuronal and developmental processes. The product of the gene is part of the enzyme phosphatase 2A, specifically the regulatory subunit B. It may regulate the catalytic activity and substrate selectivity of the enzyme [57]. Moreover, missense variants in this gene have already been associated with ASD with ID and other comorbid symptoms [58–60]. Since the carriers of the pathogenic variants in the *PPP2R5D* gene in the Slovenian cohort have similar phenotypes to those mentioned above, namely autism in combination with ID (Table 1), this supports the association of the discovered variants with their phenotypes.

Of the two variants discovered, PPP2R5D(NM_006245.4):c.758G>A (p.*Arg*253*Gln*) has not been described in association with ASD yet. This variant is classified as a variant of unknown significance (VOUS) because it meets PM2, PP2, and PP3 criteria of the ACMG guidelines [61]. The results of this case-control association study (Fig 4), and the *in silico* protein structure and function analysis (Fig 8) support a pathogenic effect of the variant. It is very rare in the GnomAD database and the amino acid substitution is semi-conservative, which, according to the I-TASSER analysis results, affects the protein function. More specifically, these results of protein structure and function prediction suggest that the mutation impacts the ligand binding function of the protein (Fig 8). The phenotype of the patient, carrying this variant, also matches the phenotypes of patients, carrying variants in the *PPP2R5D* gene. Considering all these facts, this variant might have an impact on the phenotype of autism and ID. Because the *in silico* prediction supports criteria PP3 of the ACMG guidelines, we propose to classify the PPP2R5D(NM_006245.4):c.758G>A (p.*Arg*253*Gln*) variant as likely pathogenic.

The other variant, PPP2R5D(NM_006245.4):c.592G>A (p.*Glu*198*Lys*), has already been described in the context of ID and autism [59]. It is classified as pathogenic because it meets PM1, PM2, PP2, PP3, and PP5 criteria of the ACMG guidelines [61]. The consequence of the variant is a non-conservative amino acid substitution that could affect the secondary structure of the protein. Functional studies revealed deficient formation of the protein in cells expressing this mutation [62]. Because the present study detected this pathogenic variant in association with ID and the autism phenotype, this result indicates that the presented approach is suitable to detect the probably pathogenic variants.

The combination of case-control analysis using large public databases, similar phenotypes, and the use of o/e and Z scores in this gene-burden analysis led to the discovery of probably pathogenic variants in an ASD-associated gene. These results support the usefulness of o/e and Z scores in the interpretation of patient’s variants. The results obtained also confirm that gene-burden analysis combined with appropriate filtering of data based on population frequencies, predictions of pathogenicity prediction tools, o/e, and Z scores, is a suitable tool to detect the probably pathogenic variants in genes, associated with ASD.

## Conclusions

The results of this gene-burden study show an association of two risk variants with ASD, one of which has not previously been described in association with this disorder. The results of examination of significant new variants using protein structure and function prediction software also support the pathogenic effect of the variant. This suggests that the proposed filtering and pathogenicity prediction method is suitable for detecting rare deleterious variants in sequences from ASD patients. It provides a new opportunity for clinicians to discover additional, disease-causing variants not previously described and to screen out those, already defined as pathogenic.

However, gene-burden studies using publicly available control data have several limitations. The main limitations are the number of patients included in a study and the lack of a healthy control group from the same population. To ensure better power of the analysis, researchers conducting these analyses can collect data from more patients, as well as some healthy controls. Collecting data from 100 more patients could improve the reliability of the results of the gene-burden analysis. Including a healthy control group from the studied population could provide more accurate filtering of pathogenic variants based on frequency. Regardless of whether the pathogenicity of the new variants is supported by the structure and function prediction tool results, *in vitro* analysis of the effects of the new variants could provide additional insight into changes at protein level.

## Supporting information

S1 Raw images (PDF)
