# Supplementary figures and images for: Identification of potentially pathogenic variants for autism spectrum disorders using gene-burden analysis
Source: PLoS One. 2023 May 11;18(5):e0273957. doi: 10.1371/journal.pone.0273957 (PMC10174571; doi:10.1371/journal.pone.0273957)

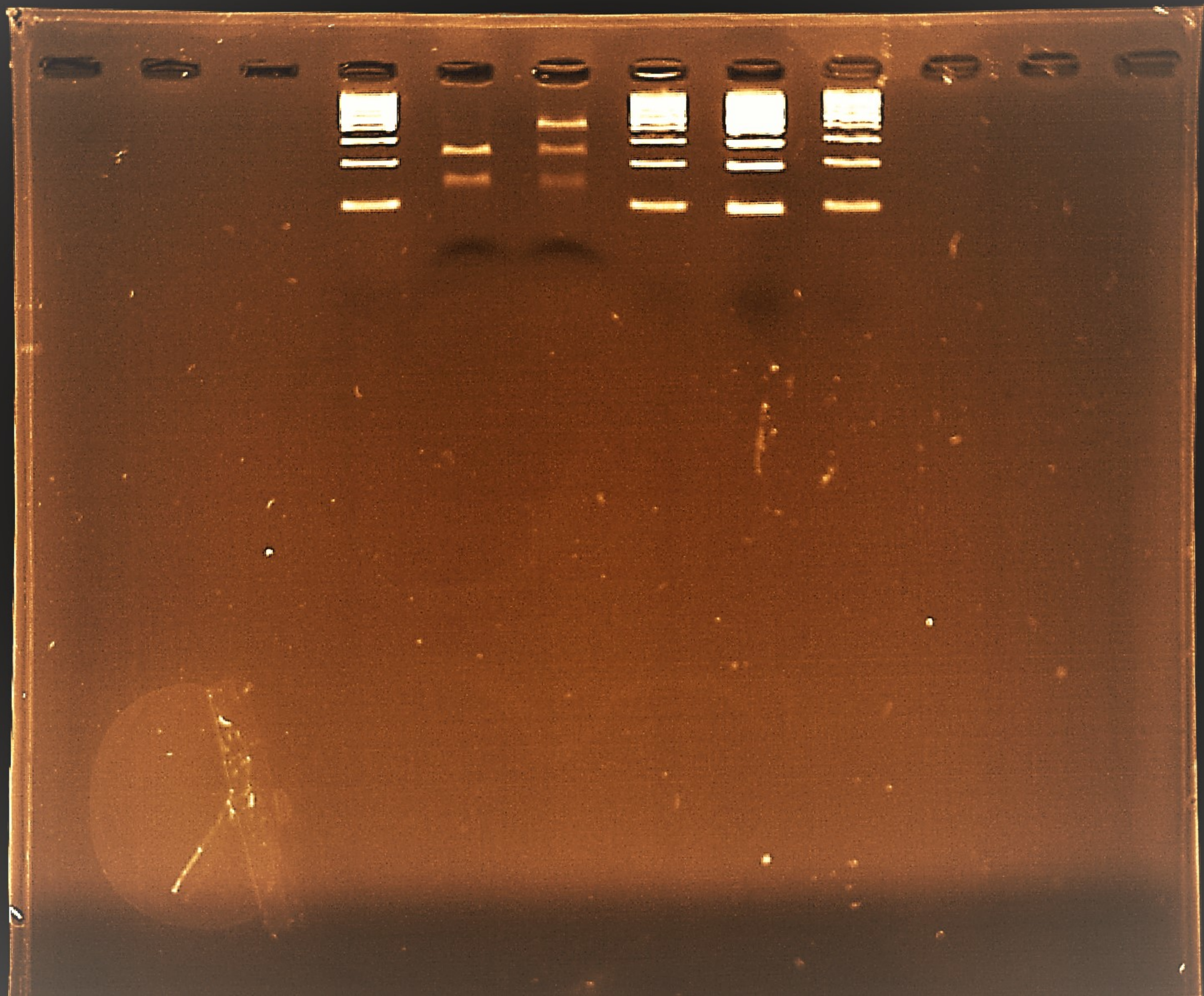

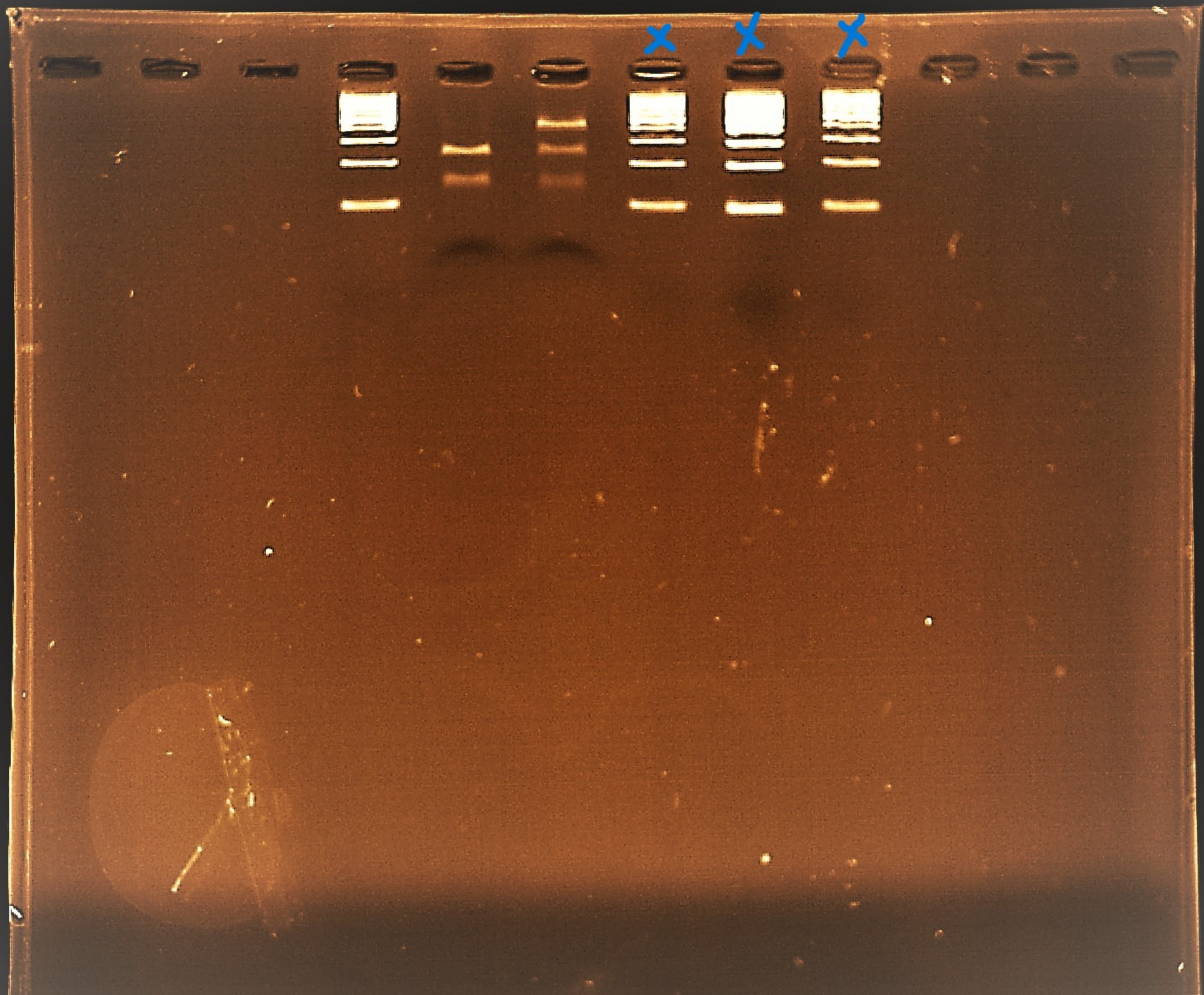

1

2

3

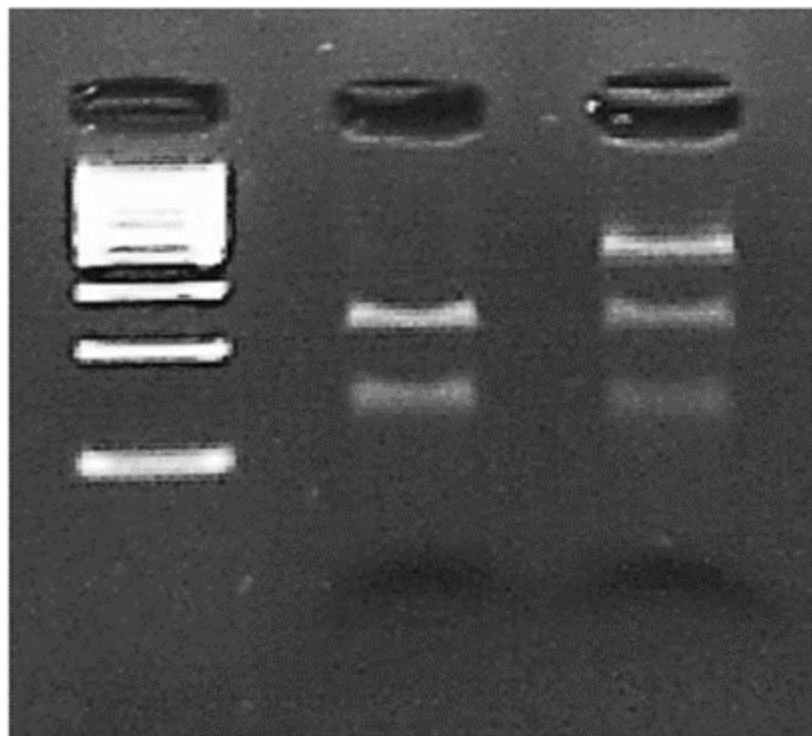

351 bp

220 bp

131 bp

Supplement: S1 Raw images — (PDF) [file pone.0273957.s001.pdf]
